# Divergences in Leaf Economic Traits Among Five Congeneric Tree Species in a Subtropical Forest

**DOI:** 10.1002/ece3.71511

**Published:** 2025-06-01

**Authors:** Qiuju Chen, Linyan Wu, Rong Li, Zhi Yin, Yinping Jiang, Yanjiao Mao, Chao Zhang, Yi Jin, Xiaoxin Tang, Yin Yi

**Affiliations:** ^1^ School of Life Sciences Guizhou Normal University Guiyang China; ^2^ Key Laboratory of National Forestry and Grassland Administration on Biodiversity Conservation in Karst Mountainous Areas of Southwestern China Guizhou Normal University Guiyang China; ^3^ Key Laboratory of Plant Physiology and Developmental Regulation of Guizhou Province Guizhou Normal University Guiyang China

**Keywords:** *Carpinus*, closely related species, karst forest, Maolan Nature Reserve, niche partitioning, sympatry

## Abstract

Exploring the differences in leaf economic traits between co‐occurring congeneric angiosperm species will advance the understandings of coexistence mechanisms of closely related species in sympatry. Here, we investigated the divergence in eight leaf economic traits and the underlying ecological drivers among 293 individual plants of five congeneric tree species of *Carpinus* (Betulaceae) that commonly co‐occur in a karst forest in southwestern China. We found there was generally a large proportion of trait variation that resided at the interspecific level, and these congeneric species commonly differed in leaf economic traits. We also found these congeneric species frequently exhibited divergent topographic habitat‐mediated trait shifts and displayed trait ranking reversals along the environmental gradients. However, these congeners did not differ in plant size‐dependent shifts of leaf economic traits. These findings imply the separation in resource‐use strategies among the congeneric species of *Carpinus*, which might contribute to resource niche partitioning and hence facilitate their coexistence in the karst forests.

## Graphical abstract

Knowledge of how congeneric species co‐occur in a community differ in leaf economic traits, will advance the understandings of factors that contribute to the coexistence of closely related species in sympatry, especially in the karst forests of southwestern China that are generally rich in biodiversities. Here, we addressed this question and found that separation in leaf economic traits, as well as divergence in habitat‐mediated shifts of leaf economic traits, commonly exist between five congeneric tree species belonging to a common genus *Carpinus* (Betulaceae) in the karst forest of Maolan Nature Reserve in southwestern China.

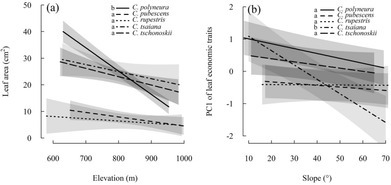

## 1 Introduction

In species‐rich ecological communities, a repeated pattern is the co‐occurrence of a number of closely related species that are similar in morphology (e.g., Valladares et al. 2000; Forrestel et al. 2015; Fortunel et al. 2020). Given that similar species will experience intense competition and hence high risk of species exclusion, it is puzzling how these closely related species that co‐occur achieve coexistence. In this context, investigating whether and how closely related species co‐occur differ in their functional traits would shed light on the understandings of coexistence mechanisms of closely related species in sympatry (Weber and Strauss 2016).

**FIGURE 1:**
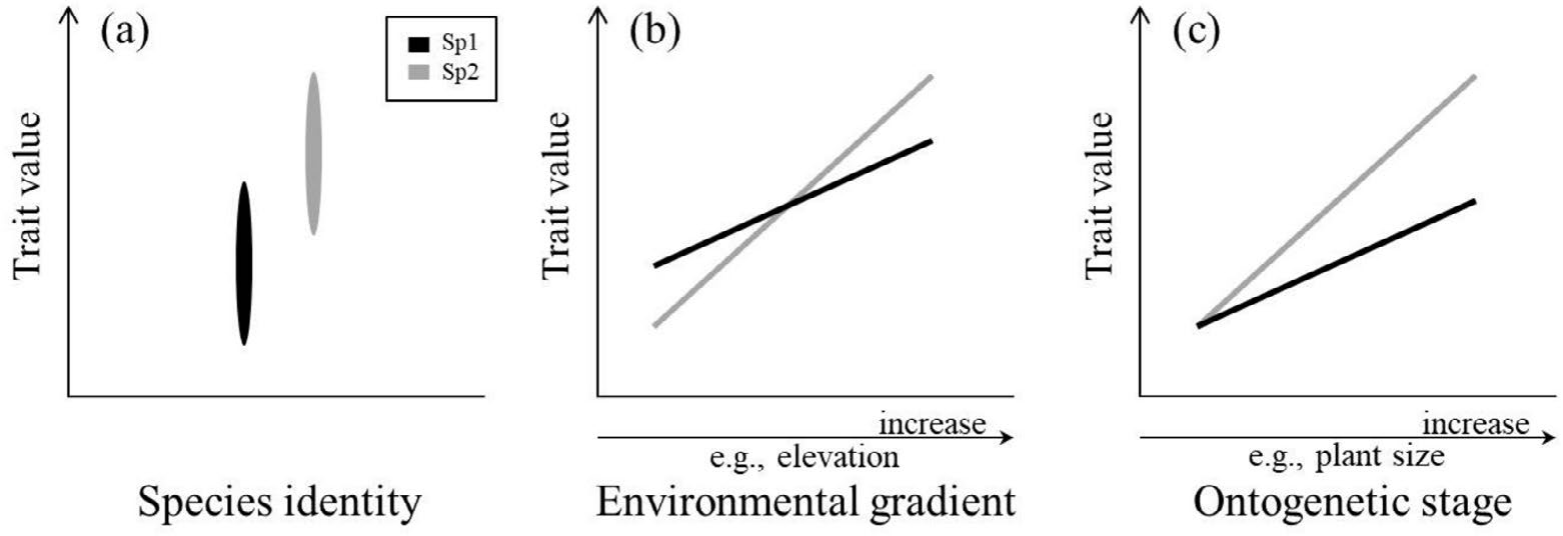
Schematics of leaf economic trait divergence between closely related species in sympatry. Species one (Sp1) and two (Sp2) are closely related species. The diagrams show, (a) Differentiation of trait value. The closely related species diverge in the values of their leaf economic traits (e.g., leaf thickness). (b) Differentiation of habitat‐mediated trait shifts. The closely related species differ in the shift of their leaf economic traits (i.e., different trait plasticity) along the environmental gradients (e.g., elevation). (c) Differentiation of ontogenetic trait trajectory. The closely related species diverge in how their leaf economic traits shift through ontogenetic stages (e.g., plant size).

In angiosperms, it is widely recognized that a set of leaf morphological and chemical traits (i.e., leaf economic traits), such as specific leaf area (SLA) and leaf nitrogen concentration, shift coordinately among plants and form a leaf economics spectrum (LES), which reflects the variation of plant resource‐use strategies (Reich 2014). For example, leaves with low construction costs and fast leaf economics (e.g., high SLA and leaf nitrogen concentration) typically reflect acquisitive resource‐use strategies; whereas the opposite traits suggest conservative strategies. Also, leaf economic traits are closely linked to habitat conditions (Reich 2014). In particular, species with leaves of acquisitive resource‐use strategies commonly reside in high resource environments, whereas species with leaves of conservative resource‐use strategies are advantageous in low resource environments (Geekiyanage et al. 2018). If closely related species differ in leaf economic traits (Figure 1a), then there possibly exists interspecific separation in resource‐use strategies (Forrestel et al. 2015), which would enhance stabilizing niche differences (Chesson 2000).

At the intraspecific level, leaf economic traits also shift with habitat conditions (Paoli 2006; Zheng et al. 2022). For example, in a hyperdiverse tropical forest of the Guiana Shield, Schmitt et al. (2020) found that the leaf economic traits of conspecifics shifted from acquisitive to conservative resource‐use strategies, with the decrease of wetness from lowlands to plateaus. Moreover, if the habitat‐mediated trait shifts (i.e., intraspecific trait plasticity) differ between species (Givnish et al. 2004; Delalandre et al. 2023), then there is probably interspecific divergence of ecophysiological strategies in coping with the resource gradients (Adler et al. 2013; Pérez‐Ramos et al. 2019; Zirbel and Brudvig 2020). Specifically, interspecific differences in trait plasticity could result in the reversal of trait rankings on the environmental gradients. And trait ranking reversal possibly leads to competitive trade‐offs and reduces fitness differences between species (Chesson 2000; Pérez‐Ramos et al. 2019; Figure 1b), and contributes to the coexistence of closely related species in sympatry.

Moreover, species could achieve coexistence via regeneration niche partitioning (Grubb 1977), such that different species show different ontogenetic trajectories of functional traits (Forrestel et al. 2015). For example, in a glasshouse experiment, Forrestel et al. (2015) found that 16 congeneric species of *Lasthenia* (Asteraceae) displayed diverse trends of leaf economic traits between life stages. Between closely related angiosperm species, divergent ontogenetic trajectories of leaf economic traits (Figure 1c), therefore, would indicate the presence of regeneration niche partitioning (Mason et al. 2013; Forrestel et al. 2015).

An extensive zone of subtropical eastern Asia is covered with karst forests (Geekiyanage et al. 2018). These forests grow on the karst landscapes (i.e., landscapes sculptured out of water‐soluble rocks such as limestone and dolomite) and are generally rich in biodiversity (Geekiyanage et al. 2019). Previous studies in these karst forests showed that topographic and edaphic factors influence the distribution of leaf economic traits both at the interspecific (Geekiyanage et al. 2018) and intraspecific (Zheng et al. 2022) levels. Also, the forest canopy exhibits influence on habitat conditions (Long et al. 2005) and hence leaf economic traits inside the karst forests (Zheng et al. 2022). In addition, a large portion of the karst forest floor consists of rocky outcrops, namely rock‐bareness, which is linked with in‐site edaphic conditions in the karst forests (Huang et al. 2009; Zhang et al. 2007). Specifically, higher rock‐bareness localities retain lower amounts of soil and exhibit greater restrictions on water and soil nutrients availabilities, which shape the spatial distribution of leaf economic traits of karst forest plants (Zheng et al. 2022).

However, the differences in leaf economic traits among closely related woody angiosperms co‐occurring in the karst forests, and the ecological drivers underlying these differences, are still poorly known. Here, in the karst forest of Maolan Nature Reserve located in subtropical southwestern China, we addressed this question by focusing on five congeneric tree species of *Carpinus* (Betulaceae). In particular, we seek to know (1) the differences in leaf economic traits between the congeneric species of *Carpinus*; (2) if these congeneric species exhibit divergent habitat‐mediated shifts in leaf economic traits; (3) if these congeneric species show divergent ontogenetic trajectories in leaf economic traits.

## 2 Materials and Methods

### 2.1 Study Sites and Species

This study is conducted in the karst forest of Maolan Nature Reserve (25°09′20″–25°20′50″ N, 107°52′10″–108°05′40″ E) located in southwestern China. Local annual mean temperature is 15.3°C, and local annual precipitation is 1752.5 mm (Zheng et al. 2022). The bedrock in the reserve consists of Carboniferous and Permian limestones and dolomites, with elevation ranges from 430 to 1078 m a.s.l. (Zhou 1987). The major geomorphology feature in the reserve is karst peak‐cluster, which is a cluster of steep hills rooted from a common base. The height from the peak‐cluster hilltop to the bottom ranges from 150 to 300 m (Li and Li 1987). Over 90% of the reserve is forested, predominantly with evergreen and deciduous broad‐leaved mixed forest (Zhou 1987).

The studied plants belong to the genus *Carpinus*, which is recognized as a monophyletic group within the family of Betulaceae (Yoo and Wen 2002). *Carpinus* comprises approximately 52 species, most of which are trees in growth form, and is widely distributed in the temperate and subtropical zones of the Northern Hemisphere (Dong et al. 2022). Specifically, *Carpinus* is a common genus in the forest of Maolan reserve (Zhou 1987). The congeneric *Carpinus* species regularly co‐occur and demonstrate extensive overlap in their topographic distributions (authors' personal oberservation).

### 2.2 Plant Sampling and Trait Measurement

We adopted the line‐transect method in sampling the plants of *Carpinus* in the karst forest of Maolan. The line extends from the bottom to the top of a hill (covering its whole elevational range) of a peak‐cluster. Nine lines were surveyed in nine peak‐clusters (hereafter referred to as sites) widely distributed in the reserve. The individuals of *Carpinus* with stem diameter at breast height (DBH, 1.3 m aboveground) ≥ 1 cm within 10 m distance from the line on both sides were sampled and investigated. A total of 293 stems were sampled, recorded, and taxonomically assigned to five species, which are *Carpinus polyneura* (92 stems), *C. pubescens* (102 stems), *C. rupestris* (41 stems), *C. tsaiana* (16 stems), and *C. tschonoskii* (42 stems). All five species are classified as deciduous broad‐leaved trees, according to Flora of China (https://www.iplant.cn/).

We measured the stem DBH of each sampled plant, using an electronic digital vernier caliper (PD‐151, Pro'sKit, Shanghai, China) for stems with DBH < 5 cm, and using a diameter tape for stems with DBH ≥ 5 cm. We measured the height of each sampled plant using a pole mounted with a rod level. For leaf sampling, we randomly sampled 10–20 fully expanded intact leaves on the outer layer at the upper crown of the plant using a tree pruner (LZ5625, VMP, Shandong, China). The sampled leaves were packed in black plastic bags, stored in portable refrigerating box at above 0°C and transported back to the laboratory the same day. Leaves were then placed in fresh water for rehydration overnight. The following day, sampled leaves were scanned using a flatbed scanner. The leaf area (LA) was measured as the sum of one‐sided projected fresh lamina surface area without petiole, using ImageJ software (Bayramzadeh et al. 2008; Schneider et al. 2012; Atar et al. 2024). For each sampled leaf, leaf thickness (LT) was measured as the mean of three measurements taken between the tip and base of the lamina, avoiding the major veins, using an electronic digital vernier caliper (PD‐151, Pro'sKit, Shanghai, China). The average LA and LT of the sampled leaves of an individual plant were used to represent the LA and LT of the plant, respectively. After that, the fresh weight of all the sampled leaves without petiole was measured in an electronic scale (BP‐223A+, SETPRO, Shanghai, China) for each individual plant. The leaves were then dried in an oven (101‐3BS, LICHEN, Shanghai, China) at 70°C for 72 h, and the dry leaf mass was measured in an electronic scale (BP‐223A+, SETPRO, Shanghai, China). SLA was calculated as the sum of LA divided by the sum of dry leaf mass of the sampled leaves for each individual plant. Leaf dry‐matter content (LDMC) was calculated as the sum of dry leaf mass divided by the sum of fresh weight of the sampled leaves for each individual plant. Leaf carbon (C) and nitrogen (N) concentrations were determined on a percent dry mass basis by elemental analyzer (vario MACRO cube, Elementar, Germany). Leaf phosphorus concentration (P) was determined on a percent dry mass basis by the Mo‐Sb antiluminosity method. Leaf C: N ratio was calculated as leaf carbon concentration divided by leaf nitrogen concentration.

### 2.3 Habitat Condition Assessment

For each sampled plant, we estimated five local habitat attributes, including elevation, slope, aspect, the neighborhood rock‐bareness rate, and canopy height. The elevation was estimated using a GPS receiver (G120BD, UniStrong, Beijing, China); slope and aspect were estimated using a geological compass. Aspect represents the direction the slope faces and is a circular variable (Legendre et al. 2009). Thus, aspect was converted with cosine(aspect) to represent the south–north facing direction, which varies between −1 and 1; a larger value of cosine(aspect) indicates a more northerly direction, following Spasojevic et al. (2014). The neighborhood rock‐bareness rate represents the percentage of forest floor composed of rocky outcrops within five meters of the sampled plant and was visually estimated to 10 levels, each representing a 10‐percentage interval from 1 (0%–10%) to 10 (90%–100%), following Zheng et al. (2022). The neighborhood forest canopy height was estimated using a measuring pole mounted with a rod level. Specifically, the height of the highest living foliage directly above the sampled plant and over four points at a five‐meter radius from the sampled plant at four random directions was estimated; the average of the five measurements was used to represent the neighborhood canopy height (Welden et al. 1991). In more than one third (i.e., 104 plants) of the 293 sampled plants, the stems were rooted on rock surfaces or from rock crevices and were in extremely high rock‐bareness neighborhoods where soil samples can not be obtained. Therefore, edaphic conditions were only estimated for the remaining 189 plants with soil samples. Specifically, the edaphic conditions were represented by five soil variables, including soil depth, soil total calcium concentration, total nitrogen concentration, total phosphorus concentration, and total carbon concentration. For each of the 189 plants, soil depths were measured, and soil samples were taken at five points within two meters distance from each individual plant. Soil sampling depth includes the 0–20 cm layer; if the soil layer is less than 20 cm, the whole layer was sampled. Soil depth of a plant was represented by the mean of the five measurements. Soil samples for each plant were well mixed and dried at room temperature; then soil total carbon, total nitrogen, and total phosphorus concentrations were measured by elemental analyzer (vario MACRO cube, Elementar, Germany), and soil total calcium concentration was measured by ICP‐OES (iCAP 7200).

### 2.4 Statistical Analyses

Principal component analysis (PCA) was performed on the eight leaf economic traits to derive a composite measure across the studied *Carpinus* species. The significance of principal components was assessed using the broken‐stick method (Jackson 1993). Meanwhile, the phylogenetic tree of the five studied *Carpinus* species was obtained by pruning the phylogeny, including 33 species of *Carpinus* from Dong et al. (2022). After that, we performed the Mantel test to examine if the divergence in leaf economic traits between the five congeneric species was constrained by phylogeny, using the “mantel” function in the *ecodist* package (Goslee and Urban 2007) in R 3.6.1 (R Core Team 2021). We found no relationship between phylogenetic distance and interspecific difference in the mean of a single leaf economic trait or trait PC1 (the first principal component from eight traits via PCA) (Mantel test: *r* = −0.359 to 0.141, *p* = 0.275–0.673). Therefore, we did not include phylogenetic relationships between the study congeneric species in the analyses.

One‐way analysis of variance was conducted using the “aov” function, to estimate the distribution of variance of each leaf economic trait and trait PC1, at the interspecific and intraspecific levels. Also, pairwise species difference in leaf economic trait and trait PC1 was estimated by Student's *t*‐test, with *p*‐values corrected by Holm's method. Moreover, a linear mixed‐effects model was conducted to estimate if habitat‐mediated shifts of leaf economic traits differ between species. In the model, the response variable was each of the eight leaf economic traits as well as trait PC1; the fixed terms of the model were individual plant size, the five habitat variables, and species identity, plus the two‐way interactions between the habitat variables and species identity. Random intercept specified in the model was site identity. Also, the linear mixed‐effects model was conducted using the “lmer” function implemented in the *lmerTest* package in R, to estimate if the size‐dependent shift in leaf economic traits differs between species. In the model, the response variable was each of the eight leaf economic traits and trait PC1; the fixed terms were individual plant size and species identity, plus their two‐way interactions. Random intercept specified in the model was site identity. Specifically, individual plant size represents the principal component one (explaining 90.1% of the variance) of stem DBH and plant height, and was adopted as a proxy of life stage following Zheng et al. (2022).

Backward selection was performed on the linear mixed‐effects models, starting with the interaction terms; the fixed terms were sequentially removed until only the fixed intercept remained. Among the candidate models, the one with the lowest AICc value (Akaike information criteria corrected for small sample size) was selected as the best‐supported model, using the “model.sel” function from the *MuMIn* package (Bartoń 2016). In model selection, AICc estimation based on maximum likelihood (ML) was adopted for fixed‐effect model comparisons. After model selection, coefficients of the best‐supported model were estimated using restricted maximum likelihood (REML). Furthermore, to investigate the post hoc pairwise species difference in habitat‐mediated shifts (for each leaf economic trait and trait PC1), each best‐supported model incorporating interaction terms was re‐executed three times, systematically changing the reference species identity using the “relevel” function. From these analyses, all 10 *p*‐values of the interaction terms corresponding to the pairwise species comparisons in the best‐supported model were extracted (Jin et al. 2018). The 10 *p*‐values from each interaction term were then adjusted for multiple comparisons using Holm's method via the “p.adjust” function.

Additionally, for the set of 189 plants with soil data, the integrated effects of topographic and edaphic habitat conditions on leaf economic traits were also estimated, following the model construction and selection procedures described above. The results (Tables S1 and S2) were consistent with the results of the analyses concerning only topographic habitat‐mediated trait shifts for the full set of 293 plants. Therefore, we only report the results of the topographic habitat‐mediated trait shifts for the full set of plants.

## 3 Results

### 3.1 Trait Differences Among Plants

According to PCA, the first principal component (trait PC1) explained 42% of the total variance in leaf economic traits and was the only significant component. Moreover, trait PC1 was positively related to SLA (Pearson's *r* = 0.74), LA (*r* = 0.71), N (*r* = 0.79) and P (*r* = 0.71) and negatively related to C: N (*r* = −0.86), LDMC (*r* = −0.4), LT (*r* = −0.38) and C (*r* = −0.33) (Figure S1). Therefore, trait PC1 was considered a composite measure of leaf economic traits across the five studied *Carpinus* species, with higher PC1 values corresponding to a faster LES.

**FIGURE 2:**
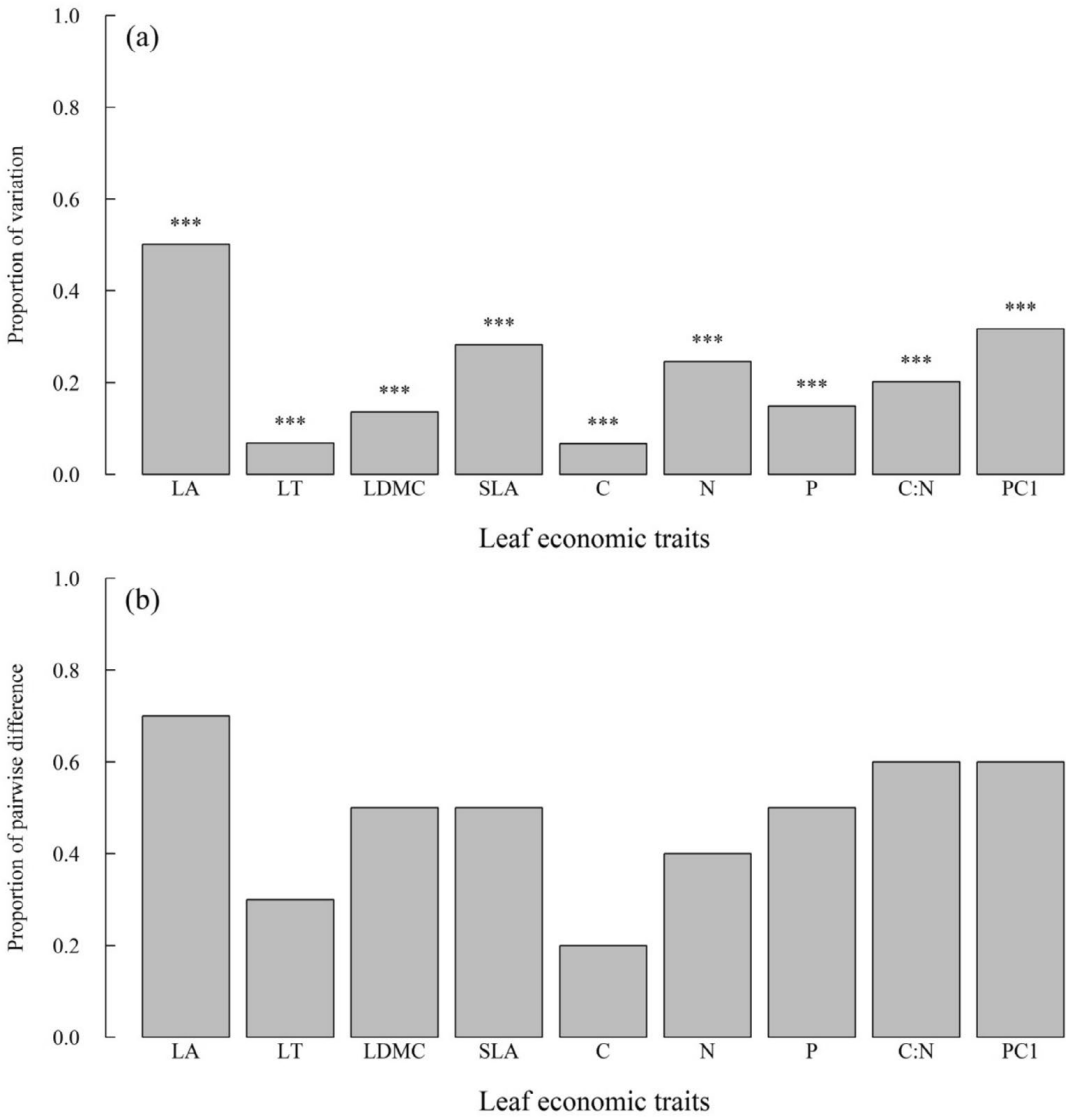
The proportion of total variation of leaf economic traits residing at the interspecific level (a), and the proportion of significant pairwise species differences in leaf economic traits (b). Significance level: ***p < 0.001. Refer to Table 2 for key to abbreviations.

There was generally a large proportion of leaf economic trait variation that resided at the interspecific level, ranging from 6.7% for leaf carbon concentration to 50.1% for LA, with an average of 20.6%. For trait PC1, 31.7% of the variation resided at the interspecific level, while the remaining 68.3% was at the intraspecific level (Figure 2a). With respect to the pairwise combinations of the five *Carpinus* species, the proportion of pairwise species trait difference (*P*_adj_ < 0.05, adjusted by Holm's method) ranged 0.20–0.70, with an average of 0.48. Among these traits, LA showed the largest proportion of 0.70 (seven out of 10 pairs), while leaf carbon concentration showed the least at 0.20 (two out of 10 pairs) (Figure 2b). On the other hand, for individual pairs of species, only two pairs did not show trait differences; the remaining eight pairs each differed (*p*_adj_ < 0.05) in four to eight leaf traits, accounting for proportions of 0.44–0.89 of the nine leaf traits (Table 1).

**TABLE 1:** The number and proportion of cases of differences in leaf economic traits for each pair of species of *Carpinus*.

	*C*. *polyneura*	*C*. *pubescens*	*C*. *rupestris*	*C*. *tsaiana*
*C*. *pubescens*	8/0.89			
*C*. *rupestris*	6/0.67	0/0		
*C*. *tsaiana*	4/0.44	4/0.44	4/0.44	
*C*. *tschonoskii*	4/0.44	7/0.78	6/0.67	0/0

*Note:* Numbers before the slash are the number of cases of differences in leaf economic traits between species, and values after the slash are the proportion of cases of differences in leaf economic traits between species.

### 3.2 Habitat‐Mediated and Plant Size‐Dependent Trait Shifts

**FIGURE 3:**
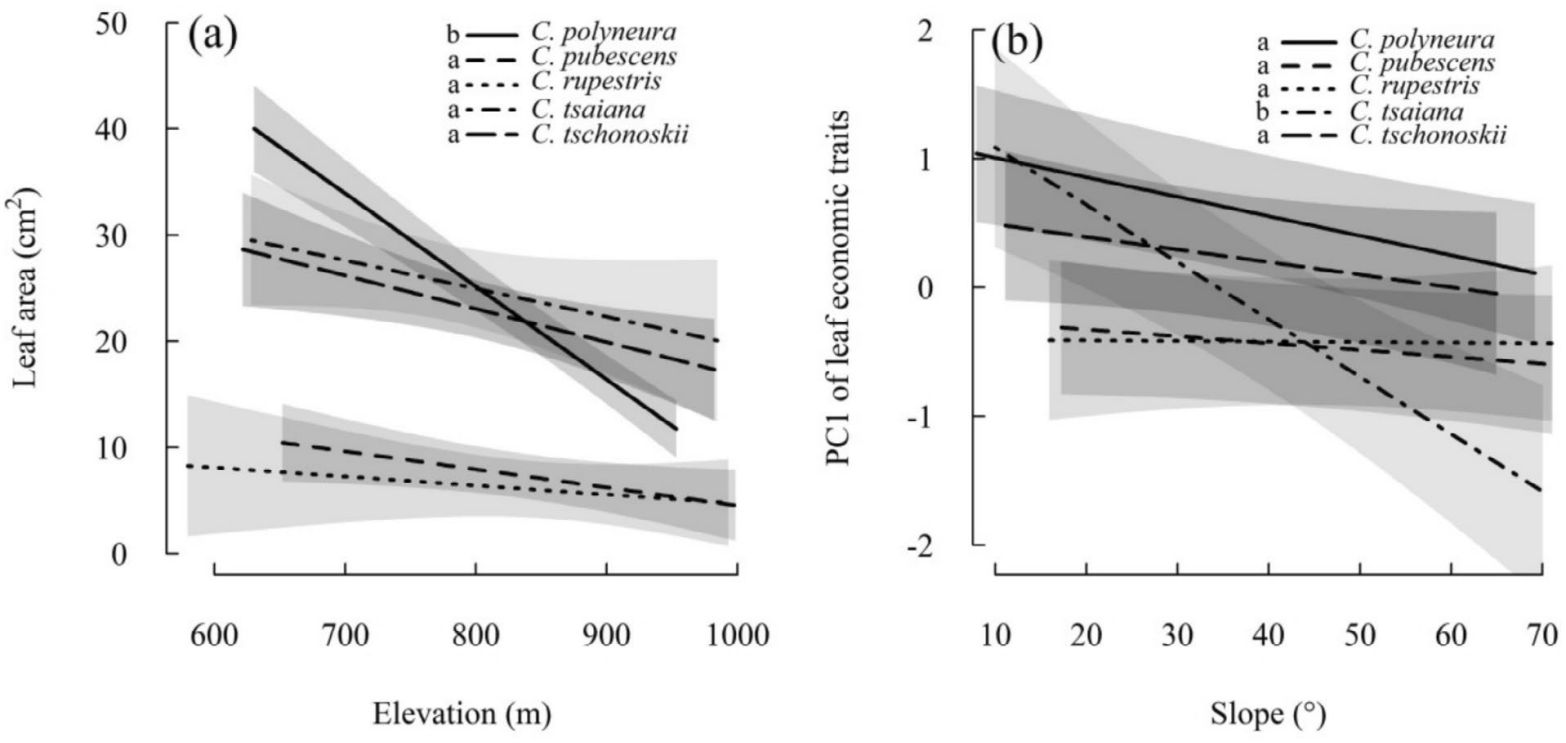
Differences among the five congeneric *Carpinus* species in their shift of leaf area along elevation gradient (a), and principal component one (PC1) of leaf economic traits along topographic slope gradient (b), as estimated by the best‐supported linear mixed‐effects models in Table 2. The lines show the predicted trait shift of the five species of *Carpinus*, and the shaded regions show 95% confidence regions for the lines. Letters on the left side of the legend denote post hoc tests of the difference in slopes between species, with different letters indicating significantly different slopes. Key to abbreviation: *C*., *Carpinus*.

Based on the best‐supported linear mixed‐effects models, the five congeneric *Carpinus* species differed in 10 cases of habitat‐mediated trait shifts (i.e., intraspecific trait plasticity; the interaction terms in Table 2). For example, with increasing elevation, LA generally decreased (toward more conservative resource‐use strategy), with the rate of decline varying among species (Figure 3a). Notably, *C. polyneura* demonstrated the most pronounced decline in LA with increasing elevation across all *Carpinus* species, along with trait ranking reversals relative to *C. tsaiana* and *C. tschonoskii* (Figure 3a). With increasing topographic slope, trait PC1 generally declined (toward more conservative resource‐use strategy), also with varied rates of decline among species. Among the five *Carpinus* species, *C. tsaiana* displayed the strongest reduction in trait PC1 with increasing topographic slope, as well as trait ranking reversals (Figure 3b). Moreover, in pairwise species comparisons, three of the 10 species pairs did not show different habitat‐mediated shifts of leaf economic trait or trait PC1, while the remaining seven pairs each exhibited differences (*p*_adj_ < 0.05) in two to eight traits (Table 3). By contrast, the five *Carpinus* species did not differ in their size‐dependent shifts of leaf economic trait or trait PC1 (no interaction terms in Table S3).

**TABLE 2:** Analysis of variance table for the best‐supported linear Mixed‐Effects model of habitat effects on the leaf economic traits.

	LA	LT	LDMC	SLA	C	N	P	C: N	PC1
Size		14.50***		22.26***			7.56**	2.26	
Ele	120.97***	8.85**	29.70***	24.29***	42.98***		55.47***	15.53***	72.10***
Slp	13.12***		1.52			7.78**	9.56**	10.62**	12.55***
Asp	17.07***		2.82			7.38**	0.31	7.84**	0.19
Roc									
CH	4.53*	11.91***	5.91*	16.90***		3.35		0.79	15.06***
Species	352.43***	34.88***	31.93***	111.57***	30.47***	98.12***	37.05***	90.69***	142.70***
Ele × Species	71.42***				13.58**				
Slp × Species	20.03***		11.64*						16.53**
Asp × Species	28.05***						11.84*		10.73*
Roc × Species									
CH × Species						12.70*		16.18**	

*Note:* Wald type III test statistics are shown for models with interaction terms; Wald type II test statistics are shown for models without interaction terms. Significance levels: *p < 0.05; **p < 0.01; ***p < 0.001. “×” indicates interaction between factors.

Abbreviations: Asp, aspect; C, leaf carbon concentration; C:N, leaf carbon to nitrogen ratio; CH, canopy height; Ele, elevation; LA, leaf area; LDMC, leaf dry‐matter content; LT, leaf thickness; N, leaf nitrogen concentration; P, leaf phosphorus concentration; PC1, principal component one of the eight leaf economic traits; Roc, rock‐bareness rate; SLA, specific leaf area; Slp, slope.

**TABLE 3:** The number of cases of difference in the habitat‐mediated shift of leaf economic traits for each pair of species of *Carpinus* as shown by the post hoc test of the best‐supported linear mixed‐effects models in Table 2.

	*C. polyneura*	*C. pubescens*	*C. rupestris*	*C. tsaiana*
*C. pubescens*	3			
*C. rupestris*	2	0		
*C. tsaiana*	2	8	3	
*C. tschonoskii*	2	0	0	2

*Note:* The total number of cases of difference (the theoretical maximum number of cases of significant difference) for each species pair is 10 (i.e., the number of interaction terms as shown in Table 2).

## 4 Discussion

We still lack a clear understanding of how co‐occurring, closely related species in species‐rich communities differ in leaf economic traits, despite the potential role of such differences in promoting their coexistence in sympatry (e.g., Pérez‐Ramos et al. 2019). In this study, we uncovered some evidence of the divergences in leaf economic traits between the congeneric species of *Carpinus*, which commonly co‐occur inside the karst forests of eastern Asian subtropics.

Generally, there was a large proportion of leaf economic trait variation that resided at the interspecific level, with frequent pairwise species trait differences between the congeneric species of *Carpinus*. This finding indicates that divergence in leaf economic traits is common among these closely related species. For example, we found six of the 10 species pairs differed in trait PC1, which is a composite measure of leaf economic traits, implying divergent leaf investment strategies among the five *Carpinus* species. In particular, species with larger trait PC1 likely exhibit faster leaf economics and bet on fast investment recoup in resource‐rich environments, whereas species with smaller trait PC1 likely bet on longer leaf duration accompanied with slow investment return under resource‐poor conditions (Reich 2014). This finding is in line with previous reports of divergences in the leaf economic traits between closely related angiosperm species in sympatry in the tropical regions (e.g., Givnish et al. 2004; Paoli 2006). Divergences in leaf economic traits among closely related species may contribute to species coexistence through enhanced stabilizing niche differences (Chesson 2000) and subsequent habitat partitioning (Toll 2023).

Moreover, these *Carpinus* species frequently exhibited divergent, topography‐driven shifts in leaf economic traits (i.e., different trait plasticity), suggesting that environmental heterogeneity within the karst forest drives divergent ecophysiological strategies among these congeneric species (Chazdon 1992; Valladares et al. 2000; Delalandre et al. 2023). Occasionally, interspecific divergence in trait plasticity led to species trait ranking reversals along topographic gradients. For example, high LA plasticity in *C. polyneura* and trait PC1 plasticity in *C. tsaiana* caused trait ranking reversals among congeners (Figure 3), indicating more flexible leaf investment strategies along elevation and topographic slope gradients, respectively. Given that leaf economic traits mediate competitive ability (Violle et al. 2009; Kunstler et al. 2016), these species potentially exhibit competitive trade‐offs with their congeners (Pérez‐Ramos et al. 2019). Consequently, these competitive trade‐offs diminish average fitness differences between species (Chesson 2000), thereby facilitating coexistence among closely related species in the karst forest. This finding further suggests that the congeneric *Carpinus* species exhibit fine‐scale partitioning of the environmental gradients over comparable environmental ranges (Givnish et al. 2004; Laughlin and Messier 2015), in addition to maintaining distinct optimal environmental conditions as mentioned earlier.

Forrestel et al. (2015) found divergent ontogenetic shifts in leaf traits of the congeners of *Lasthenia* in a common garden glasshouse experiment. However, we did not detect different trajectories of size‐dependent shifts of leaf economic traits between the study species of *Carpinus* in the Maolan karst forest. Therefore, our result does not support the hypothesis of regeneration niche partitioning between these congeneric species. We suspect it might partly be due to the fact that we only included the life stages of plants with DBH ≥ 1 cm, hence ignored the earlier life stages during which regeneration niche partitioning could happen (Reader et al. 1993; Larson et al. 2020). Otherwise, the possibility of inherent developmental constraints in driving differential ontogenetic trajectories between closely related species also can not be ruled out (Fortunel et al. 2020).

Taken together, we detected the divergences in leaf economic traits and topographic habitat‐mediated trait plasticity among the five congeneric *Carpinus* species in the karst forest of Maolan reserve, southwestern China. These trait differences likely enhance stabilizing niche differences and reduce fitness differences among the species (Chesson 2000), thereby promoting their coexistence in sympatry. Over evolutionary timescales, these trait patterns may reflect competition‐driven character displacement (Meilhac et al. 2020) and other selective pressures, likely contributing to *Carpinus*'s successful radiation in eastern Asia (Xue et al. 2020). In particular, such traits may have enabled the lineage to spread widely across the region's highly heterogeneous subtropical karst forests (Geekiyanage et al. 2019). Further studies are needed to uncover how the interspecific divergences in leaf economic traits contribute to the performance and long‐term coexistence (e.g., Sterck et al. 2011) of these closely related species in the karst forests.

## Author Contributions

**Qiuju Chen:** data curation (equal), writing – original draft (equal). **Linyan Wu:** data curation (equal), writing – original draft (equal). **Rong Li:** data curation (equal), writing – original draft (equal). **Zhi Yin:** writing – original draft (equal). **Yinping Jiang:** writing – original draft (equal). **Yanjiao Mao:** data curation (equal), formal analysis (equal), investigation (equal), writing – original draft (equal). **Chao Zhang:** resources (equal), writing – original draft (equal). **Yi Jin:** conceptualization (equal), data curation (equal), funding acquisition (equal), project administration (equal), writing – original draft (equal). **Xiaoxin Tang:** resources (equal), writing – original draft (equal). **Yin Yi:** funding acquisition (equal), resources (equal), writing – original draft (equal).

## Conflicts of Interest

The authors declare no conflicts of interest.

## Supporting information


Data S1.

Data S2.

## Data Availability

The data used in this study are included as a supporting Information with this article.
