# Supplementary material for: Divergences in Leaf Economic Traits Among Five Congeneric Tree Species in a Subtropical Forest
Source: Ecol Evol. 2025 Jun 1;15(6):e71511. doi: 10.1002/ece3.71511 (PMC12127140; doi:10.1002/ece3.71511)
Supplement: Supplementary file 2 — Data S2. [file ECE3-15-e71511-s002.docx]

**Supplementary Material**

**Table S1** Analysis of deviance table for the best-supported linear mixed-effects model of topographic and edaphic habitat effects on the leaf economic traits among 189 plants of five congeneric *Carpinus* species. Wald type III test statistics are shown for models with interaction terms; Wald type II test statistics are shown for models without interaction terms. Key to abbreviations: Ele, elevation; Slp, slope; Asp, aspect; Roc, rock-bareness rate; CH, canopy height; Soil_PC1, principal component one of edaphic conditions; Soil_PC2, principal component two of edaphic conditions; LT, leaf thickness; LA, leaf area; SLA, specific leaf area; LDMC, leaf dry-matter content; C, leaf carbon concentration; N, leaf nitrogen concentration; P, leaf phosphrous concentration; C : N, leaf carbon to nitrogen ratio; PC1, principal component one of the eight leaf ecnomic traits. ‘×’ indicates interaction between factors. Significance levels, *, P < 0.05; **, P < 0.01; ***, P < 0.001.

|  | LA | LT | LDMC | SLA | C | N | P | C : N | PC1 |
| --- | --- | --- | --- | --- | --- | --- | --- | --- | --- |
| Size |  | 8.88** |  | 11.38*** |  |  | 3.06 | 2.59 |  |
| Ele | 69.56*** | 7.14** | 8.59** | 9.38** | 21.15*** | 2.79 | 34.84*** | 8.37** | 53.24*** |
| Slp | 14.38*** |  | 0.50 |  |  | 12.08*** | 7.89** | 13.18*** | 13.32*** |
| Asp | 6.99** |  | 3.57 |  |  | 5.82* | 0.48 | 8.03** |  |
| Roc |  |  |  |  |  |  |  |  |  |
| CH | 2.49 | 4.21* | 5.03* | 4.92* |  | 0.86 | 1.56 | 0.12 | 3.98* |
| E1 |  |  |  |  | 4.27* |  |  |  |  |
| E2 |  |  | 1.24 |  |  |  | 4.35* |  | 2.72 |
| Species | 200.78*** | 27.79*** | 17.53** | 69.47*** | 29.60*** | 69.43*** | 16.54** | 41.02*** | 87.66*** |
| Ele × Species | 42.69*** |  |  | 9.72* | 22.15*** |  |  |  |  |
| Slp × Species |  |  | 10.11* |  |  |  |  |  |  |
| Asp × Species | 14.62** |  |  |  |  |  | 10.17* |  |  |
| Roc × Species |  |  |  |  |  |  |  |  |  |
| CH × Species |  |  |  |  |  | 10.42* | 13.81** | 14.92** |  |
| Soil_PC1 × Species |  |  |  |  |  |  |  |  |  |
| Soil_PC2 × Species |  |  |  |  |  |  |  |  |  |

Note: As high correlation exists between the soil variables (e.g., Pearson’s *r* = 0.97 between soil total carbon and total nitrogen concentrations), a principal component analysis was conducted on the five soil variables. The first two principal components (i.e., soil PCs 1 and 2) respectively explained 54% and 19% of total variance of the five soil variables, and were extracted to represent the edaphic conditions. After that, the linear mixed-effects model was conducted to estimate if topographic and edaphic habitat-mediated shifts of leaf economic traits differ between species for the 189 plants. In the model, the response variable was each of the eight leaf economic traits or trait PC1; the fixed terms of the model were individual plant size, the five aforementioned topographic habitat variables plus the two edaphic habitat variables (i.e., soil PCs 1 and 2) and species identity, plus the two-way interactions between each of these seven habitat variables and species identity. Random intercept specified in the model was site identity. After that, model selection, post-hoc pairwise species comparison for the best supported models, as well as *P*-value adjustments were conducted as mentioned in the section of ‘Statistical analyses’.

**Table S2** The number of cases of difference in the topographic and edaphic habitat-mediated shift of leaf economic traits for each pair of species of *Carpinus* as shown by the post-hoc test of the best-supported linear mixed-effects models in Table 2. The total number of cases of difference (the theoretical maximum number of cases of significant difference) for each species pair is nine (i.e., the number of interaction terms as shown in Table S1).

|  | *C. polyneura* | *C. pubescens* | *C. rupestris* | *C. tsaiana* |
| --- | --- | --- | --- | --- |
| *C. pubescens* | 3 |  |  |  |
| *C. rupestris* | 1 | 1 |  |  |
| *C. tsaiana* | 2 | 3 | 0 |  |
| *C. tschonoskii* | 2 | 0 | 0 | 0 |

**Table S3** Analysis of deviance table for the best-supported linear mixed-effects model of size-dependent effects on leaf economic traits. Wald type II test statistics are shown. Key to abbreviations: LA, leaf area; SLA, specific leaf area; LDMC, leaf dry-matter content; C, leaf carbon concentration; N, leaf nitrogen concentration; P, leaf phosphrous concentration; C : N, leaf carbon to nitrogen ratio. ‘×’ indicates interaction between factors. Significance levels, *, P < 0.05; **, P < 0.01; ***, P < 0.001.

|  | LA | LT | LDMC | SLA | C | N | P | C : N | PC1 |
| --- | --- | --- | --- | --- | --- | --- | --- | --- | --- |
| Plant size |  | 4.820* |  | 5.552* | 2.401 |  | 10.216** | 5.033* |  |
| Species | 284.6*** | 22.396*** | 47.675*** | 112.992*** | 27.729*** | 116.28*** | 48.611*** | 114.029*** | 145.15*** |
| Plant size × Species |  |  |  |  |  |  |  |  |  |

**
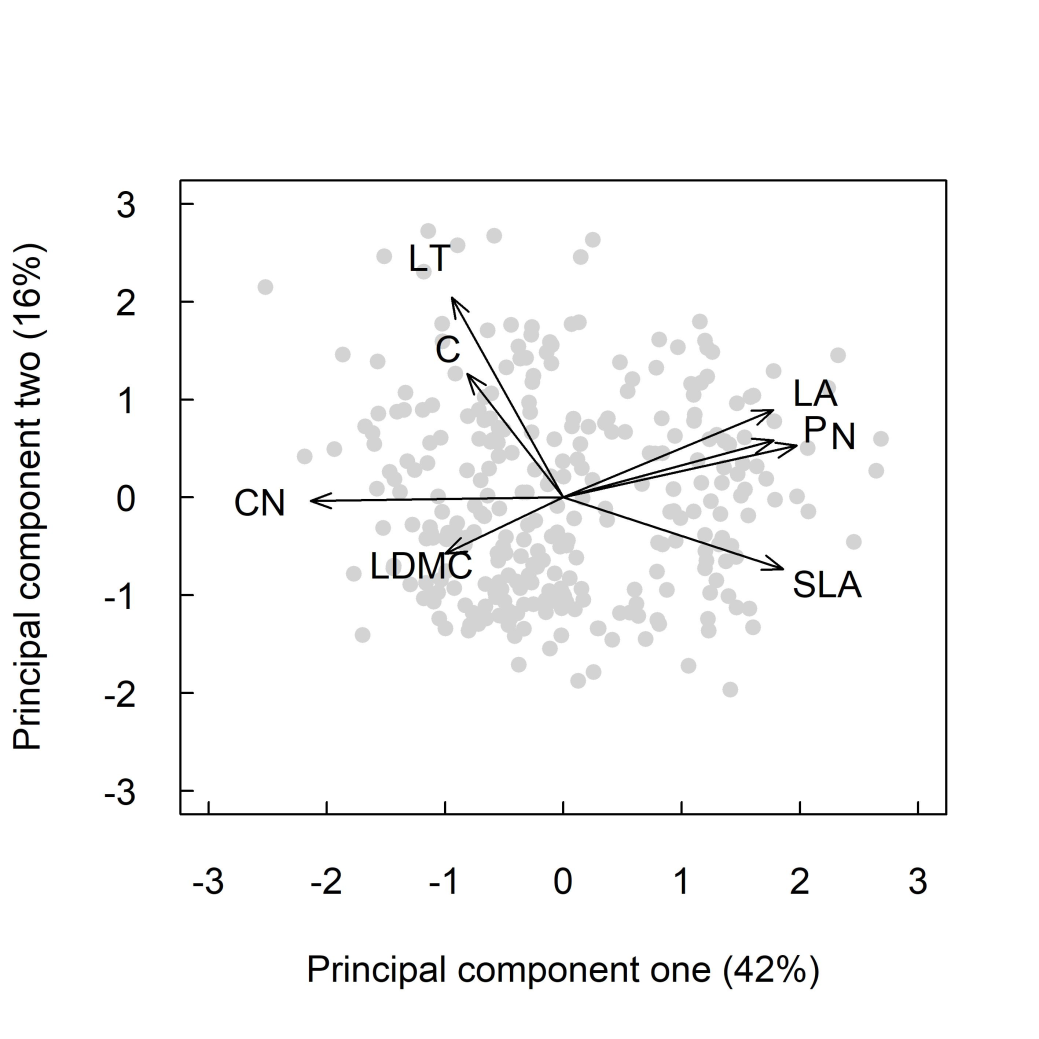
**

**Figure S1** Principal component plots of the leaf economic traits of the 293 *Carpinus* plant individuals. Key to abbreviations: LT, leaf thickness; LA, leaf area; SLA, specific leaf area; LDMC, leaf dry-matter content; C, leaf carbon concentration; N, leaf nitrogen concentration; P, leaf phosphrous concentration; CN, leaf carbon to nitrogen ratio.
